# Spelling acquisition in children through interleaved practice: the role of instructional guidance

**DOI:** 10.1186/s41235-025-00680-z

**Published:** 2025-10-09

**Authors:** Marina Klimovich, Tobias Richter

**Affiliations:** Department of Psychology IV, University of Würzburg, Röntgenring 10, 97070 Würzburg, Germany

## Abstract

We report the results of a preregistered classroom experiment (https://aspredicted.org/x25h-d427.pdf) investigating the immediate and long-term effects of interleaved practice for learning spelling rules among German third graders (*N* = 147). The study also investigated whether instructional guidance—comprising prompts and explanations that highlight key features and direct attention to relevant differences between concepts—enhances the effectiveness of interleaving by supporting comparison processes. Children completed two training sessions practicing words governed by specific spelling rules (capitalization, single and multiple consonants, words with “i” and “ie,” and words with “ss” and “ß”), with one session conducted in a blocked and the other in an interleaved format. Children made fewer spelling errors on words practiced under the interleaved condition compared to the blocked condition, both immediately after training and at an 8-week follow-up. Instructional guidance influenced performance on new, unpracticed words governed by the same spelling rules. However, its effectiveness was moderated by children’s prior knowledge: instructional guidance supported children with low prior knowledge during blocked practice in the immediate posttest and benefited children with high prior knowledge during interleaved practice at follow-up. These findings suggest that interleaved practice is an effective strategy for promoting lasting learning of spelling rules and facilitating transfer, though primarily among children with high prior knowledge. Future research should investigate whether providing children with a broader knowledge base through explicit instruction before the practice phase can help children with low prior knowledge to benefit more fully from interleaving.

## Significance statement

When learning new concepts, such as different spelling rules, learners often study multiple examples, such as words illustrating each spelling rule, to extract the underlying regularities associated with each concept. The sequence in which these examples are presented—either grouped by concept (i.e., blocked) or mixed across concepts (i.e., interleaved)—can significantly influence learning outcomes. Furthermore, learners may benefit from the combination of interleaved practice with instructional guidance, such as explanations that highlight key features and direct attention to relevant differences between concepts.

The present study investigated the effects of interleaved practice for German third graders learning similar and easily confusable spelling rules. Children who practiced words in an interleaved compared to blocked sequence showed fewer spelling errors in an immediate posttest and an 8-week follow-up test, highlighting its potential to enhance long-term retention. Furthermore, the results emphasize the added value of combining interleaved practice with instructional guidance: children with high prior knowledge made fewer spelling errors on novel words at the 8-week follow-up, indicating successful internalization of the spelling rules.

These findings suggest that interleaved practice can promote lasting and transferable knowledge. However, the emergence of transfer effects depended on children’s prior knowledge, indicating that instructional guidance alone may not be sufficient to fully support children with low prior knowledge in complex learning tasks. Future research should examine whether providing explicit instruction to build a broader knowledge base before practice can enable children with low prior knowledge to benefit more fully from interleaving.

## Introduction

In nearly all academic domains, concepts and rules are introduced through explicit instruction, but their mastery often depends on extensive practice with closely aligned examples. Research has demonstrated that simply changing the order in which examples are presented can improve learning performance (Carvalho & Goldstone, 2015; Kang & Pashler, 2012; Mitchell et al., 2008). Two common sequencing strategies are *blocking*, in which examples from the same concept are grouped together (e.g., a_1_a_2_a_3_b_1_b_2_b_3_c_1_c_2_c_3_), and *interleaving*, in which examples from different concepts are mixed (e.g., a_1_b_1_c_1_a_2_b_2_c_2_a_3_b_3_c_3_). Research suggests that interleaving is particularly beneficial for learning concepts that are highly similar and therefore difficult to discriminate (for a meta-analysis, see Brunmair & Richter, 2019). According to the sequential attention theory (Carvalho & Goldstone, 2015, 2017), this benefit arises because interleaving draws learners’ attention to the features that distinguish one concept from another. In contrast, blocking is more effective when concepts are easily distinguishable and within-category examples share minimal similarities.

Meta-analyses (Brunmair & Richter, 2019; Firth et al., 2021) have shown that relative to blocking, interleaving results in an average medium-sized effect size. However, most studies relied on a narrow range of learning materials, such as categorizing paintings by artist (e.g., Kang & Pashler, 2012; Kornell & Bjork, 2008). Moreover, most interleaving studies have been conducted in laboratory settings with adult participants, limiting their ecological validity. Only a few studies have examined interleaving in naturalistic contexts or its effects on lasting learning (Klimovich & Richter, 2025; Nemeth et al., 2021; Schweppe et al., 2025; Ziegler & Stern, 2014)—that is, learning that leads to knowledge that is available over longer periods such as several weeks after the learning episode.

In a preregistered classroom study (https://aspredicted.org/x25h-d427.pdf), we developed a spelling training program to examine whether interleaved practice, compared to blocked practice, enhances the acquisition of similar and easily confusable spelling rules in third-grade children. To our knowledge, only one previous study has examined interleaving in the context of spelling acquisition. Klimovich and Richter (2025) found that third-grade children made significantly fewer spelling errors on trained words following interleaved compared to blocked practice in an immediate posttest. At an 8-week follow-up, benefits extended to both trained and near-transfer words—but only for children with average to high prior knowledge. These findings indicate that interleaved practice supports short-term learning for all children, but those with low prior knowledge struggle to consolidate the underlying rules and show no long-term or transfer benefits.

We therefore investigated whether *instructional guidance* aimed at supporting comparison and discrimination processes could enhance the benefits of interleaved practice and increase the number of children who profit from it. Explicit prompts that encourage children to contrast exemplars may facilitate the identification of relevant features and the internalization of the underlying spelling rules. For instance, Ziegler et al. (2018) found that interleaving mathematical tasks was more effective than blocking when combined with instructional supports such as self-explanation and comparison prompts, with effects lasting up to ten weeks. However, research on instructional guidance to sustain the benefits of interleaving has primarily focused on mathematics (e.g., Nemeth et al., 2019, 2021; Ziegler & Stern, 2014). The present study aimed to extend the work of Klimovich and Richter (2025) by conducting a new investigation that additionally varied instructional guidance to examine its effect on presentation format.

Our spelling training targeted four spelling rules commonly taught in Grade 3 in Germany: (1) capitalization at the beginning of words, (2) long vs. short vowels before single vs. multiple consonants, (3) words with “i” vs. “ie,” and (4) words with “ss” or “ß.” These spelling phenomena are challenging to acquire due to their high between-category similarity, a condition under which interleaving is expected to be particularly effective. For instance, confusion between “i” (short vowel sound) and “ie” (long vowel sound, as in English “ē”) arises from their similar phonetic properties, with the critical distinction hinging on vowel length. Additionally, the syllable-based approach suggests that “ie” is typically used when the “i” sound occurs at the end of a syllable (e.g., “m*ie*-ten,” /ˈmiːtən/ [to rent]), whereas “i” is often followed by a hard consonant in the syllable coda (“m*i*t-ten,” /ˈmɪtən/ [middle]). Using these spellings correctly requires children to internalize rules of syllable structure. Detailed descriptions of the spelling phenomena and the specific challenges they pose can be found on the Open Science Framework (https://osf.io/jtzvm).

We expected that interleaved sequencing of exemplars in spelling training would outperform blocked sequencing, both for spelling performance in words trained in the sessions (Hypothesis 1) and in other words that incorporate the same spelling rules (Hypothesis 2; near transfer) immediately after the training session. Furthermore, we expected instructional guidance to enhance the positive effects of interleaved practice for both trained words (Hypothesis 3) and in near-transfer words (Hypothesis 4). We expected that the advantages of interleaved practice would persist over an 8-week follow-up for both trained (Hypothesis 5) and near-transfer words (Hypothesis 6), and that instructional guidance would continue to support performance in both trained (Hypothesis 7) and near-transfer words (Hypothesis 8) over this delayed period.

Interleaving is often considered a desirable difficulty (Bjork, 1994), that is, an instructional approach that stimulates cognitive processes beneficial for long-term retention (Richter et al., 2022; Ziegler & Stern, 2014) but that is perceived as more difficult and less effective than blocking (Yan et al., 2016, 2017). In the present study, we aimed to provide further support for the link between interleaving and desirable difficulties by assessing children’s metacognitive judgments of the learning process. We expected that children would perceive practicing exemplars in interleaved sequence as more difficult (Hypothesis 9) and rate the learning process as less successful (Hypothesis 10) compared to a blocked sequence. We also explored whether instructional guidance moderates the effect of interleaving vs. blocking on metacognitive judgments of learning.^1^

## Method

This study investigated the effects of interleaved practice on spelling acquisition in German third-grade children. It aimed to replicate a previous study by Klimovich and Richter (2025) and extend it by additionally varying instructional guidance. Accordingly, some descriptions below are identical to those in Klimovich and Richter (2025). The present study was conducted with a new and independent sample of participants.

### Power

Assuming a medium effect size (*f* = .25) for the interaction between presentation format and instructional guidance, a sample of 46 students would be required (with α = .05, 1-β = .90). However, to ensure the same level of power for the hypothesized contrasts of training condition and instructional guidance, a sample size of 81 would be required, assuming an effect size of *d* = 0.40 for each comparison (based on the average interleaving effect found in the meta-analysis by Brunmair & Richter, 2019 and the minimum desirable effect of instructional interventions according to Hattie, 2009). We deliberately targeted a sample size of 100 participants to account for attrition and other forms of data loss and for lower power because of possible clustering effects. Expecting that not all children would receive parental permission, we preregistered to recruit 250 children. The sample size was computed with G*Power (Faul et al., 2007).

### Participants

Participants were 147 children from third grade (74 girls, 69 boys, 4 missing values) with an average age of 9.37 years (6 missing values, *SD* = 0.89), drawn from ten classes across five schools in Lower Franconia (Bavaria), Germany. Parents completed a questionnaire in which they provided sociodemographic information. All children spoke German, and 27 of them spoke an additional language. Parental consent was obtained for all participating children.

### Procedure

**Fig. 1:**
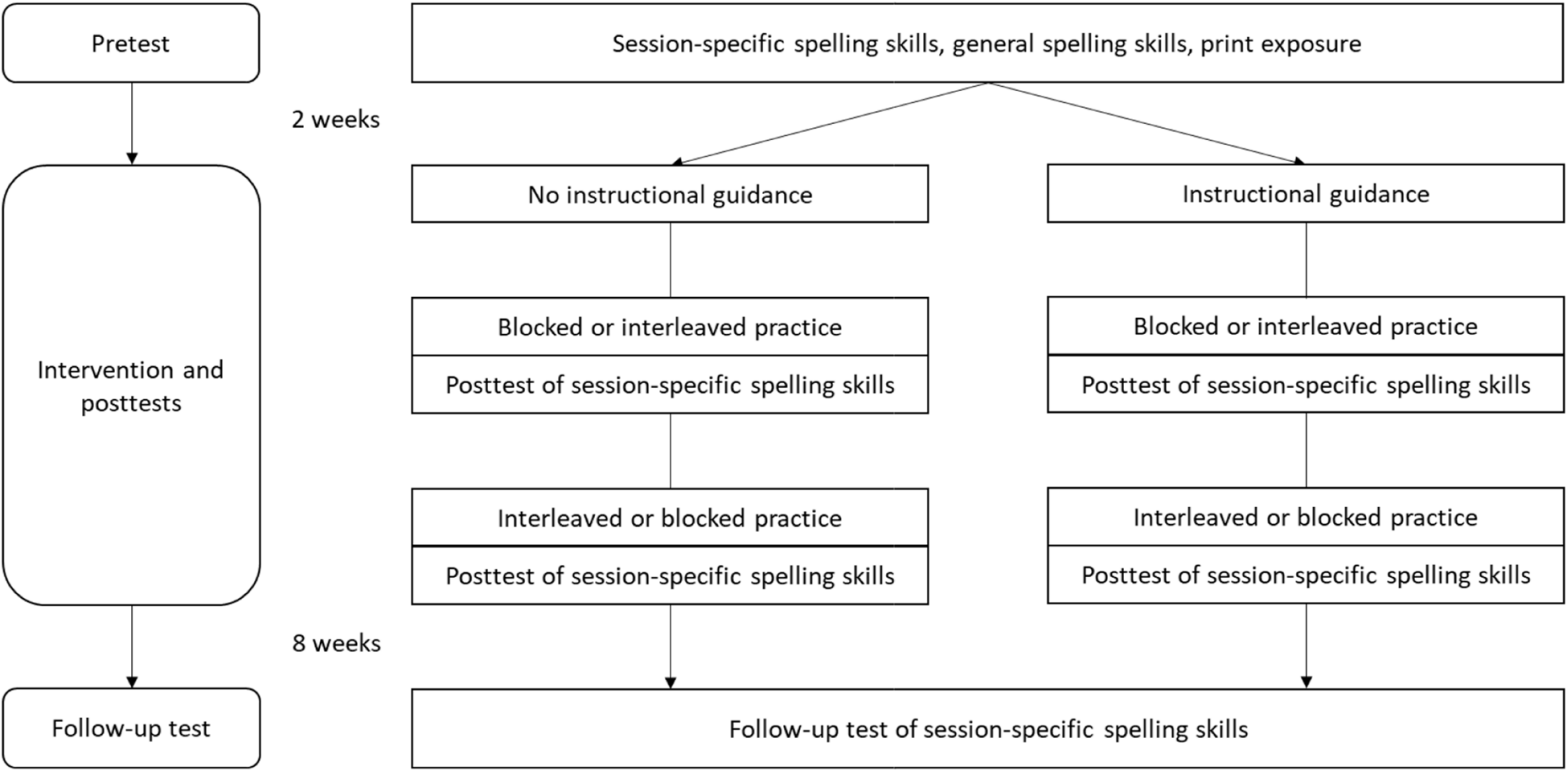
Design

This experimental study involved a pretest, an intervention phase with an immediate posttest, and a follow-up test (Fig. 1). The interval between the pretest and the intervention phase with immediate posttest was two weeks. The follow-up test was conducted eight weeks after the final training session. During the 45-min pretest, we measured (1) prior knowledge of word spellings that corresponded with the spelling rules trained in the two training sessions, (2) children’s spelling skills with a standardized spelling error detection task (Endlich et al., 2020), and (3) print exposure (KTR-T, Kinder-Titelrekognitionstest [Title recognition test for children], Schroeder et al., 2016).

In the intervention phase, all children completed two training sessions within the same week—one in the blocked format and one in the interleaved format. For example, a child might learn the rules “words with ‘i’” and “words with ‘ie’” in a blocked session, followed by the rules “words with ‘ss’” and “words with ‘ß’” in an interleaved session. The order of these formats was counterbalanced across participants. Assignment to the instructional guidance conditions was between subjects, meaning that each child completed both sessions either with or without instructional guidance. Each of the two training sessions lasted 45 mins. Following each training session, the children were asked to rate their learning difficulty, their learning success, and whether they had learned and practiced the words carefully on 5-point Likert scales. After the training session, children completed the 45-min posttest that included a session-specific error detection task (on a laptop) and a dictation task (on paper). The 45-min follow-up test assessed the learning outcomes of both conducted training sessions eight weeks later.

The study was computer-based and conducted in a classroom setting. Each child received a convertible laptop with a 15-inch screen and headphones for listening to the instructions. Responses were entered via touchscreen. No teacher involvement was required. Trained student assistants received detailed instructions on how to welcome the children, conducted the dictation tasks, and provided technical assistance during the study. Except for the dictation tasks, all materials were presented via laptops.

### Training

We developed four computerized training sessions, each covering two different spelling phenomena and following the same two-phase structure. In Phase 1, children were introduced to the spelling rules through an instructional video that featured verbal explanations and examples. In Phase 2, children practiced words related to the selected spelling rules through various tasks, such as gap-filling and identifying correct rhymes. For example, for the spelling rules “words with ‘i’” and “words with ‘ie,’” during blocked sequencing children first practiced only words with “i” (e.g., Sp*i*nne [spider], St*i*ft [pen], Bl*i*tz [flash], Br*i*lle [glasses]) and then only words with “ie” (e.g., fl*ie*gt [flies], Sp*ie*gel [mirror], s*ie*ben [seven], St*ie*fel [boots]). In contrast, during interleaved sequencing children practiced words from both rules in a mixed order (e.g., Sp*i*nne, fl*ie*gt, St*i*ft, Sp*ie*gel, Bl*i*tz, s*ie*ben, Br*i*lle, St*ie*fel).

During this practice phase, children received four prompts (two per exercise). In the *non-guided* versions, they listened to a repetition of the relevant spelling rules but were not explicitly prompted to compare specific features of the words (e.g., “Remember: when you hear a short* i* sound, you usually write* i*. When you hear a long* i* sound, you usually write *ie*. Another way to check is by breaking the word into syllables. If the *i* is not at the end of a syllable, you write a simple *i*. If the *i* is at the end of a syllable, you write *ie*.”).

In the *guided* versions, by contrast, prompts directed children’s attention to key aspects of the last two practiced words that were essential for applying the given spelling rule (e.g., “In the word *Spinne*, you write an* i*. But in the word *fliegt*, you write *ie*. Why is that? Compare the two rules.“). These last two practiced words were presented simultaneously to facilitate direct comparison and reduce the need to recall previous words from working memory (Richter et al., 2022). Children were then required to actively identify the rule-relevant feature by answering a multiple-choice question (e.g., regarding vowel length). After responding, they received an explanation of the correct answer and, as in the non-guided versions, listened to a repetition of the rules—either one rule in the blocked condition or two different rules in the interleaved condition.

**Fig. 2:**
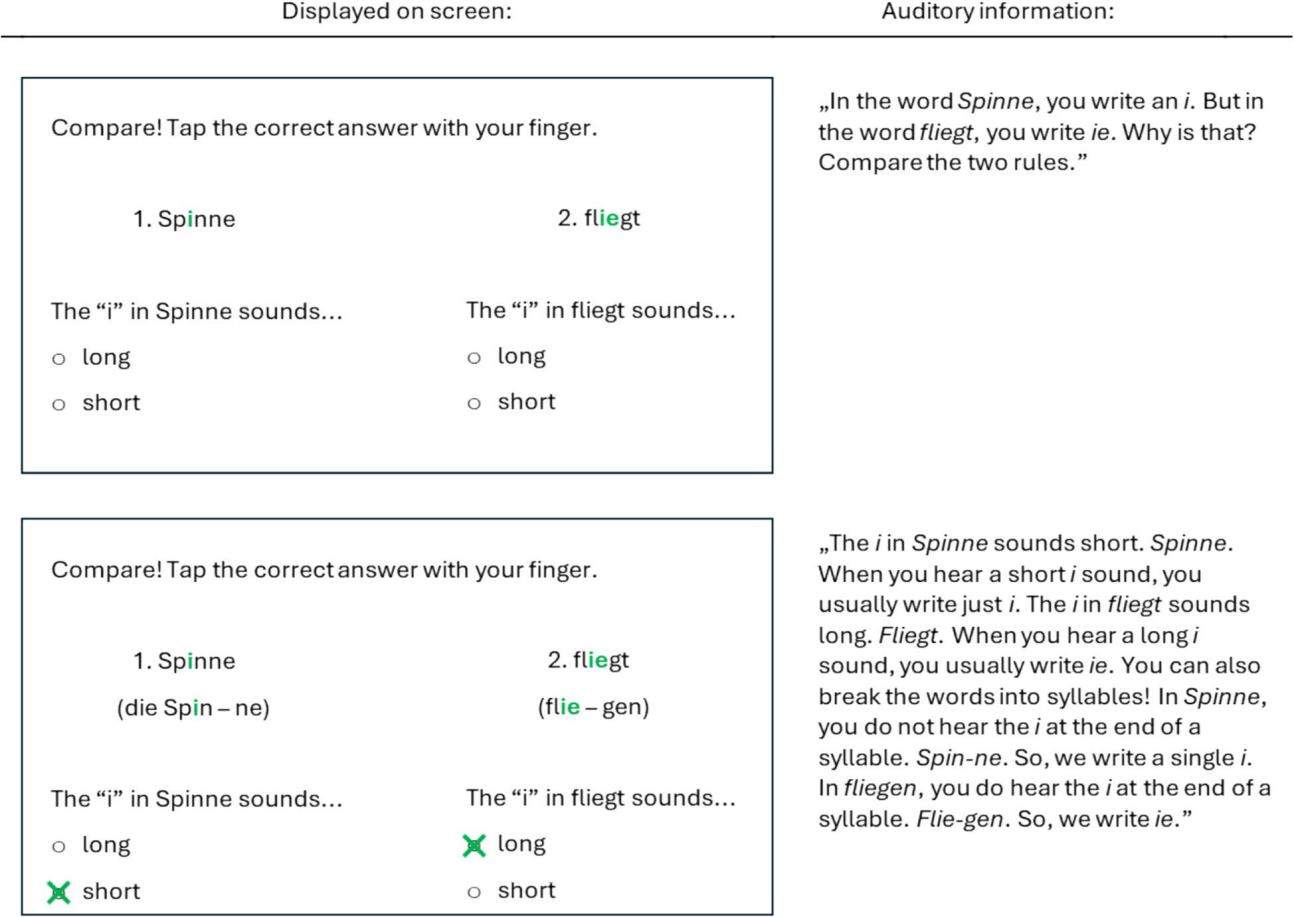
Example of how instructional guidance was implemented in combination with interleaved practice, using the spelling rules “Words with ‘i’” and “Words with ‘ie’” *Note* The prompts displayed the last two practiced words simultaneously. Children were asked to identify the rule-relevant feature by answering multiple-choice questions. Afterward, they listened to explanations of the correct answers and to a repetition of the two spelling rules. The on-screen instructions and auditory information shown here are translations from German into English

The prompts were developed according to the defining characteristics of each rule (e.g., for the rules “words with ‘i’” and “words with ‘ie,’” vowel length and a syllable-based approach are key to distinguishing between them). Figure 2 presents an example of how instructional guidance was implemented in combination with interleaved practice, using the spelling rules “words with ‘i’” and “words with ‘ie.’” Further examples regarding the combinations between instructional guidance (present vs. absent) and study sequence (blocked vs. interleaved) are available at the Open Science Framework repository (https://osf.io/jtzvm).

All materials were based on the spelling training program *Würzburger orthografisches Training* (Würzburg orthographic training, Berger et al., 2009), and the sessions were implemented using Labvanced software (Finger et al., 2017).

### Dependent variables

#### Session-specific spelling performance

To assess session-specific prior knowledge and learning outcomes in post- and follow-up tests, we developed error detection and short dictation tasks, with one of each per spelling phenomenon. The posttests and follow-up tests included separate scores for trained words and near-transfer words (governed by the same rule but not explicitly practiced). The score for the error detection tasks was always determined by subtracting the sum of correctly identified misspelled words (i.e., hits) from the number of actual misspelled words plus the number of false alarms (i.e., correctly spelled words marked as incorrect, false alarms) (see Endlich et al., 2020; Klimovich & Richter, 2025). The score for the dictation task was determined by the number of misspelled target words.

Previous studies have reported high correlations between error detection tasks and dictations (Endlich et al., 2020, 2021; Klimovich & Richter, 2025; Lenhart et al., 2019; Schneider et al., 2014), validating the notion that both types of tasks serve as equally valid indicators of spelling ability. In line with our preregistration and the approach taken by Klimovich and Richter (2025), we combined scores from the error detection and dictation tasks when their correlation reached at least .70. In the present study, the correlation between the two task types was .70, justifying their aggregation into a single composite score. This procedure yielded total scores ranging from 0 to 24 for the pretest, 0 to 24 for trained words and 0 to 24 for near-transfer words on the immediate posttest, and 0 to 24 for trained words and 0 to 24 for near-transfer words on the follow-up test. A score of 0 always indicated the best spelling performance (i.e., no spelling errors), whereas a score of 24 reflected the poorest performance. Detailed information on the materials and scoring procedure is available via the Open Science Framework repository (https://osf.io/jtzvm).

#### Metacognitive judgements of the learning process

After each training session, children rated aspects of their learning experience using 5-point Likert scales, including perceived learning difficulty (“How difficult was it for you to learn and practice the spelling of the words?”) and predicted learning success (“How well will you remember the correct spelling of the words you just learned and practiced?”).

### Design

The experiment was based on a 2 (instructional guidance: yes vs. no) × 2 (presentation format: blocked vs. interleaved) × 2 (measuring time: posttest vs. 8-week follow-up) design with instructional guidance varied between-subjects and presentation format and measuring time varied within-subjects. Children completed the training sessions either with instructional guidance (*n* = 81) or without instructional guidance (*n* = 66). All children completed two training sessions, one in the blocked format and one in the interleaved format, with the order of these two formats being counterbalanced across participants.

## Results

The analyses reported in this section were conducted as preregistered (https://aspredicted.org/x25h-d427.pdf), unless indicated otherwise.

Prior to analyses, data were screened for outliers based on model-derived standardized residuals. Observations with residuals exceeding ± 2.5 *SD* were excluded, as such deviations from model’s predicted values can disproportionately influence parameter estimates. For spelling performance, outlier removal affected 1.78% of data points for trained words and 0.72% for near-transfer words in the immediate posttest, and 1.42% for both in the follow-up test. For metacognitive judgments, 1.95% (perceived learning difficulty) and 2.33% (predicted learning success) of data points were excluded. Although not preregistered, this step enhances model robustness and validity by reducing the impact of extreme, model-inconsistent observations (Cohen et al., 2013).

We applied linear mixed models for spelling performance and metacognitive judgements of the learning process. Children and classes were included as random effects (Baayen et al., 2008) to account for the multilevel structure (children nested in classes) of the data. Instructional guidance (not guided = -1, guided = 1) and presentation format (blocked = -1, interleaved = 1) were contrast-coded and included as fixed effects. Prior knowledge of word spellings assessed at pretest was *z*-standardized and included as a condition-specific covariate. Spelling performance was always assessed by counting spelling errors; thus, lower scores indicated better performance, and higher scores reflected poorer performance (ranging from 0 to 24 spelling errors). The directed hypotheses were tested with one-tailed tests and the exploratory analyses were tested with two-tailed tests at a significance level of α < .05. Only higher-order interactions are interpreted.

In Germany, curricula can differ between states (Bundesländer) but are uniform within each state. Since all children were recruited from schools in Lower Franconia, they followed the same curriculum. Before the pretest, between the pretest and posttest, and between the posttest and the 8-week follow-up teacher reports on covering the selected rules were solicited. According to these reports, the rules had been taught in 20% of cases before the pretest, in 25% of cases between the pretest and posttest, and in 10% of cases between the posttest and follow-up. During the training sessions, children generally reported that they had learned and practiced the words carefully (*M* = 4.53, *SD* = 0.68).

All data, the reproducible R code, and analyses based on the full dataset (without the exclusion of outliers) are available at the repository of the Open Science Framework (https://osf.io/jtzvm).

### Spelling performance

#### Immediate posttest

**Fig. 3:**
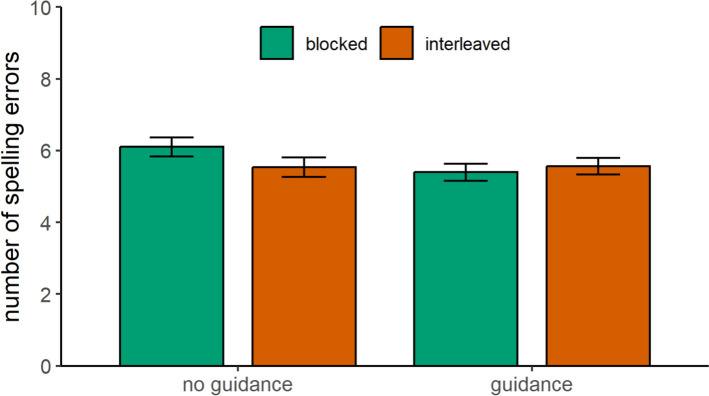
Spelling performance of trained words at the immediate posttest *Note* Spelling performance of trained words was estimated as a function of instructional guidance and presentation format. Spelling performance is measured by the total number of spelling errors. Therefore, lower scores indicate better performance. Error bars show standard errors of the mean

Full model results can be found in Table 1. For trained words, the interaction effect between presentation format and instructional guidance was significant (Fig. 3). Pairwise comparisons revealed that, in the absence of instructional guidance, children made fewer spelling errors when they practiced words in an interleaved format compared to a blocked format, β = 0.56, *SE* = 0.33, *t*(62.8) = 1.70,* p* = .047. However, when instructional guidance was provided, no significant difference was found between the blocked and interleaved presentation formats, β = -0.16, *SE* = 0.28, *t*(41.7) = -0.58,* p* = .565.

**Table 1:** Parameter estimates for spelling performance of trained words and near-transfer words at immediate posttest

Predictors	Trained words					Near-transfer words				
	β	*SE*	*t*	*df*	*p*	β	*SE*	*t*	*df*	*p*
Intercept	5.64	0.15	36.43	10.12	< .001	6.47	0.17	37.69	9.36	< .001
Presentation format	0.10	0.11	0.92	119.79	.179	-0.01	0.14	-0.06	243.53	.474
Guidance	0.17	0.13	1.35	119.68	.178	0.10	0.14	0.74	248.58	.462
Prior knowledge	2.03	0.12	16.75	222.79	< .001	2.31	0.14	16.12	248.68	< .001
Presentation format × guidance	0.18	0.11	1.67	119.80	.048	0.21	0.14	1.49	243.75	.069
Presentation format × prior knowledge	0.00	0.11	0.04	181.70	.966	-0.08	0.14	-0.56	246.42	.578
Guidance × prior knowledge	-0.00	0.12	-0.01	222.11	.989	0.03	0.14	0.21	250.11	.833
Presentation format × guidance × prior knowledge	0.01	0.11	0.11	182.03	.910	0.42	0.14	2.95	246.26	.003

**Fig. 4:**
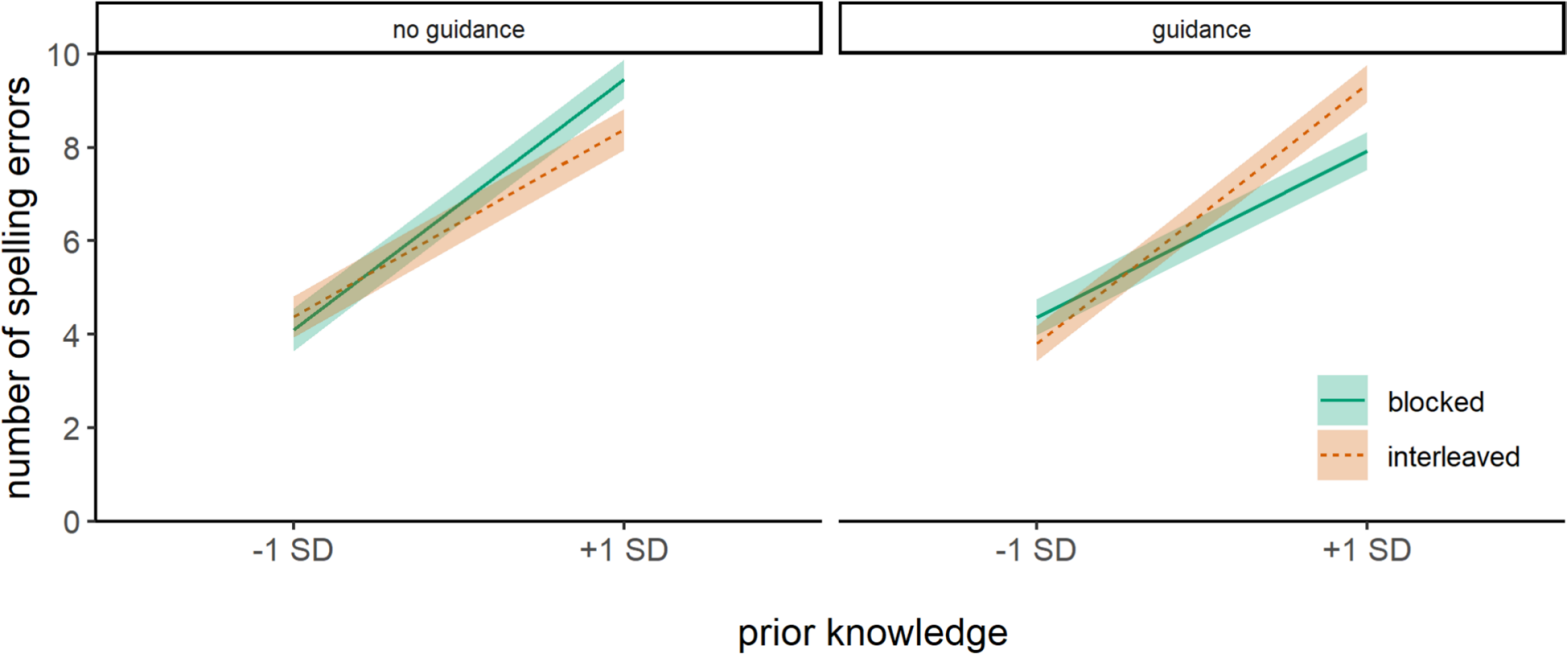
Spelling performance of near-transfer words at the immediate posttest *Note* Spelling performance of near-transfer words was estimated as a function of prior knowledge, presentation format, and instructional guidance. Spelling performance is measured by the total number of spelling errors. Therefore, lower scores indicate better performance. Shaded areas around each line represent standard errors

For near-transfer words, we found a significant three-way interaction effect between prior knowledge, presentation format, and instructional guidance (Fig. 4). An analysis of conditional effects revealed that in the absence of instructional guidance, there was no significant effect of presentation format in children with high (-1 *SD*), β = -0.28, *SE* = 0.62, *t*(155.9) = -0.44,* p* = .659, or low (+ 1 *SD*), β = 1.08, *SE* = 0.58, *t*(132.9) = 1.86,* p* = .065, prior knowledge. When instructional guidance was provided, no significant effect of presentation format was found for children with high prior knowledge (-1 *SD*), β = 0.56, *SE* = 0.52, *t*(120) = 1.08,* p* = .284. However, children with low prior knowledge (+ 1 *SD*) made fewer spelling errors when they had practiced words in a blocked format than in an interleaved format, β = -1.44, *SE* = 0.55, *t*(130.8) = -2.61,* p* = .010, when instructional guidance was provided.

In sum, children benefited from interleaved practice for trained words in the absence of instructional guidance, providing support for Hypothesis 1. However, we found no evidence for an interleaving benefit on near-transfer words in the immediate posttest. Thus, Hypothesis 2 was not supported. Contrary to our expectations, instructional guidance did not enhance the effects of interleaved practice for either trained or near-transfer words. Therefore, Hypotheses 3 and 4 were not supported. Instead, instructional guidance was particularly beneficial for children with low prior knowledge when practicing near-transfer words in a blocked format (Exploratory Analysis).

#### Follow-up test after 8 weeks

**Fig. 5:**
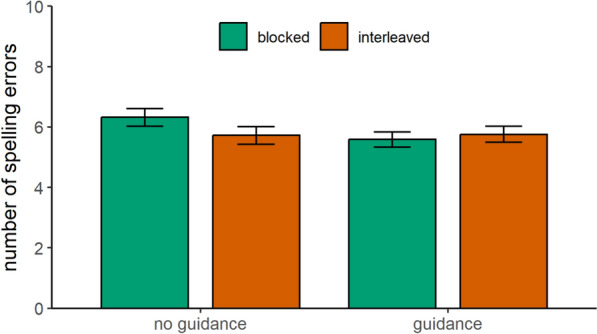
Spelling performance of trained words at the follow-up test *Note.* Spelling performance of trained words was estimated as a function of instructional guidance and presentation format. Spelling performance is measured by the total number of spelling errors. Therefore, lower scores indicate better performance. Error bars show standard errors of the mean

Full model results can be found in Table 2. For trained words, the interaction effect between presentation format and instructional guidance was significant (Fig. 5). Pairwise comparisons revealed that, in the absence of instructional guidance, children made fewer spelling errors when they had practiced words in an interleaved format compared to a blocked format, β = 0.60, *SE* = 0.32, *t*(54.1) = 1.90,* p* = .032. However, when instructional guidance was provided, no significant difference was found between the blocked and interleaved presentation formats, β = -0.17, *SE* = 0.28, *t*(34.6) = -0.60,* p* = .550.

**Table 2:** Parameter estimates for spelling performance of trained words and near-transfer words at 8-week follow-up test

Predictors	Trained words					Near-transfer words				
	β	*SE*	*t*	*df*	*p*	β	*SE*	*t*	*df*	*p*
Intercept	5.86	0.17	33.62	10.12	< .001	5.85	0.15	40.02	122.70	< .001
Presentation format	0.11	0.11	1.02	102.28	.167	0.07	0.12	0.60	114.05	.276
Guidance	0.17	0.15	1.17	110.81	.245	0.46	0.15	3.14	122.70	.002
Prior knowledge	2.02	0.14	14.93	127.24	< .001	2.21	0.14	15.94	208.24	< .001
Presentation format × guidance	0.19	0.11	1.83	102.07	.035	-0.04	0.12	-0.37	114.05	.357
Presentation format × prior knowledge	0.03	0.11	0.23	136.18	.819	-0.16	0.12	-1.30	156.90	.197
Guidance × prior knowledge	-0.08	0.14	-0.61	217.32	.545	0.25	0.14	1.79	208.24	.075
Presentation format × guidance × prior knowledge	0.14	0.11	1.25	136.42	.215	0.38	0.12	3.06	156.90	.002

**Fig. 6:**
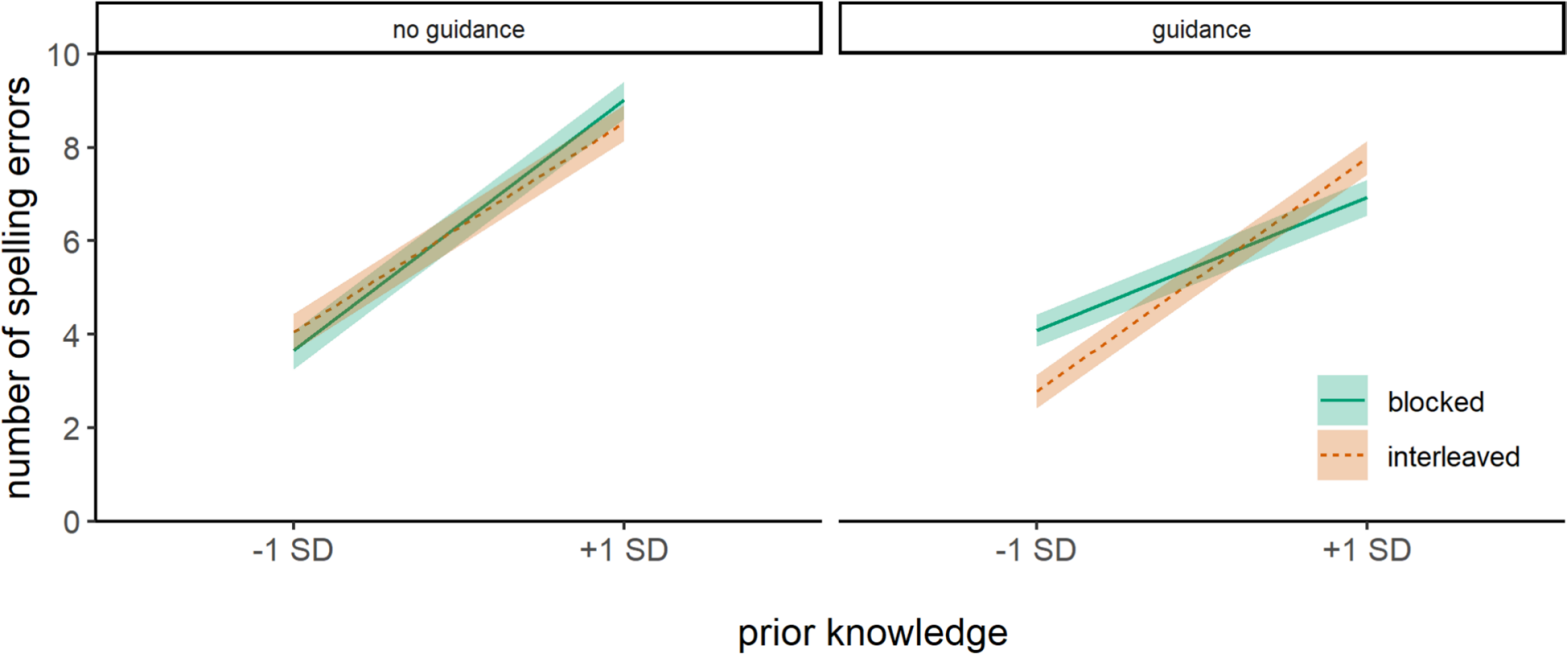
Spelling performance of near-transfer words at the follow-up test *Note.* Spelling performance of near-transfer words was estimated as a function of prior knowledge, presentation format, and instructional guidance. Spelling performance is measured by the total number of spelling errors. Therefore, lower scores indicate better performance. Shaded areas around each line represent standard errors

For near-transfer words, the three-way interaction effect between prior knowledge, presentation format, and instructional guidance was significant (Fig. 6). The analyses of conditional effects revealed that in the absence of instructional guidance, there was no significant effect of presentation format in children with high (-1 *SD*), β = -0.38, *SE* = 0.51, *t*(142.3) = -0.76,* p* = .451, or low (+ 1 *SD*), β = 0.49, *SE* = 0.50, *t*(144.8) = 0.98,* p* = .330, prior knowledge. However, when instructional guidance was provided, children with high prior knowledge (-1 *SD*) made fewer spelling errors when they practiced words in an interleaved format than in a blocked format, β = 1.30, *SE* = 0.44, *t*(122.2) = 2.94,* p* = .004, whereas no significant effect of presentation format was found for children with low prior knowledge (+ 1 *SD*), β = -0.85, *SE* = 0.48, *t*(118.7) = -1.77,* p* = .080.

In sum, the 8-week follow-up test revealed that children benefited from interleaved practice on trained words in the absence of instructional guidance, providing support for Hypothesis 5. In contrast, we found no evidence for an interleaving benefit on near-transfer words in the follow-up test in the absence of instructional guidance. Thus, Hypothesis 6 was not supported. Instructional guidance did not enhance the effects of interleaved practice for trained words, providing no support for Hypothesis 7. However, for near-transfer words, we found a delayed benefit of interleaved practice when instructional guidance was provided, but only for children with high prior knowledge. This finding offers partial support for Hypothesis 8, since this effect was moderated by prior knowledge (Exploratory Analysis).

### Metacognitive judgements of the learning process

Full model results can be found in the repository of the Open Science Framework (https://osf.io/jtzvm).

#### Perceived learning difficulty

We found no significant main effect of presentation format, β = -0.02, *SE* = 0.03, *t*(120.93) = -0.74,* p* = .229, and no significant interaction effect between presentation format and instructional guidance, β = 0.01, *SE* = 0.03, *t*(120.93) = 0.31,* p* = .377. The three-way interaction between prior knowledge, presentation format and instructional guidance was also not significant, β = -0.02, *SE* = 0.03, *t*(176.94) = -0.64,* p* = .524. Thus, Hypothesis 9 was not supported.

#### Predicted learning success

The main effect of presentation format was not significant, β = 0.01, *SE* = 0.03, *t*(118.27) = 0.34,* p* = .367. However, there was a significant interaction effect between presentation format and instructional guidance, β = 0.08, *SE* = 0.03, *t*(118.25) = 2.45,* p* = .008. Pairwise comparisons revealed that in the absence of instructional guidance, children predicted a higher learning success when they practiced words in a blocked format (*M* = 4.56, *SE* = 0.10) compared to an interleaved format (*M* = 4.38, *SE* = 0.10), β = 0.18, *SE* = 0.10, *t*(52.6) = 1.84,* p* = .036. Thus, Hypothesis 10 was supported. However, when instructional guidance was provided, no significant difference was found between the blocked (*M* = 4.28, *SE* = 0.08) and interleaved (*M* = 4.42, *SE* = 0.08) presentation formats, β = -0.14, *SE* = 0.09, *t*(36.5) = -1.62,* p* = .114. The three-way interaction between prior knowledge, presentation format and instructional guidance was not significant, β = 0.04, *SE* = 0.04, *t*(157.55) = 1.26,* p* = .209.

## Discussion

This study investigated whether interleaved practice more effectively promotes the acquisition of spelling rules in third-grade children than blocked practice, and whether instructional guidance enhances the effectiveness of interleaving by supporting comparison processes.

Results showed that, without instructional guidance, children who practiced words in an interleaved format made fewer spelling errors on trained words—both immediately and after an 8-week delay. These findings align with prior research on interleaving (Carvalho & Goldstone, 2017; Kang & Pashler, 2012; Mitchell et al., 2008; Ziegler & Stern, 2014; Ziegler et al., 2018) and further support its long-term benefits for spelling acquisition in third-grade children (Klimovich & Richter, 2025). Notably, instructional guidance did not further enhance interleaving benefits for trained words. This suggests that the juxtaposition of similar exemplars likely prompted spontaneous comparison processes, enabling learners to identify and differentiate critical features—sufficient to at least support the retention of trained words even without explicit instructional support.

Although unguided interleaving improved performance on trained words, it did not support transfer to novel words. In contrast, instructional guidance facilitated transfer, but its effectiveness depended on learners’ prior knowledge and the practice format. Specifically, children with low prior knowledge benefited from guidance only when practice followed a blocked sequence. For these children, interleaved practice—even with guidance—may have exceeded their cognitive capacity, whereas blocked practice combined with instructional guidance likely reduced cognitive demands and helped them apply the targeted rules during the immediate posttest. However, these gains did not persist over time, consistent with prior findings that blocked practice primarily supports short-term performance (Bjork & Bjork, 2011; Rohrer & Taylor, 2007). The absence of interleaving benefits for near-transfer words may suggest that, although interleaving facilitated correct spelling of trained words, the transfer of underlying spelling rules to novel words requires more time for consolidation (Klimovich & Richter, 2025).

Indeed, children with high prior knowledge showed delayed transfer effects when interleaved practice was combined with instructional guidance. This finding aligns with Klimovich and Richter (2025), who also reported delayed—but not immediate—transfer effects of interleaving. However, unlike the present study, those effects emerged without guidance, possibly due to differences in task complexity. In the current study, the target spelling phenomena involved distinctions between “ss” and “ß.” Distinguishing these spelling rules are particularly challenging, as they rely on vowel length distinctions rather than phonemic differences in the “s” sound, thereby increasing the demands on learners’ ability to identify rule-relevant features. In this context, instructional guidance likely played a crucial role in directing attention to relevant features and enabling effective comparison during interleaved practice (e.g., Nemeth et al., 2019, 2021; Ziegler & Stern, 2014). Results on the effects of the four spelling phenomena on spelling performance are available on the Open Science Framework (https://osf.io/jtzvm).

From a theoretical perspective, these findings are largely in line with the sequential attention theory (Carvalho & Goldstone, 2015, 2017), which posits that interleaving enhances learning when categories are difficult to discriminate, as the format encourages learners to attend to subtle differences between exemplars. The spelling rules targeted in the present study involved such difficult-to-distinguish categories, rendering them particularly amenable to interleaved learning. Furthermore, the results are broadly consistent with the notion that interleaved practice constitutes a desirable difficulty. Although presentation format had no significant effect on perceived difficulty, children rated their learning success as lower following interleaved compared to blocked practice in the absence of instructional guidance. This finding aligns with previous research indicating that effortful yet effective learning strategies, such as interleaving, often lead to underestimations of learning success (Bjork & Bjork, 2011; Kornell & Bjork, 2008; Yan et al., 2016, 2017). When instructional guidance was provided, this effect disappeared, suggesting that the influence of guidance on predicted learning success outweighed the impact of practice format.

From a practical perspective, an important consideration concerns the implementation of instructional guidance in the present study. The training program was computer-based, with each child working individually on their own computer. Guidance was thus delivered uniformly to all participants (i.e., the same number of prompts), ensuring comparability between conditions—a level of standardization that would be difficult to achieve if guidance were provided by a teacher in a group setting. In addition, children did not merely listen to prompts; they actively responded to multiple-choice questions targeting rule-relevant features and received immediate, individualized feedback on their answers. Immediate feedback has been shown to enhance students’ learning outcomes (e.g., Wisniewski et al., 2019) but providing it to each child individually is often not feasible in typical teacher-led lessons. However, unlike real child–teacher interactions, the guidance in the training program of the present study could not adapt in real time to provide additional follow-up support when a child required further clarification. Developing more fine-grained, adaptive digital guidance may therefore represent a promising approach to further enhance the positive effects of interleaving on children’s learning outcomes.

### Conclusion and future directions

Overall, the present findings indicate that interleaved practice is an effective approach for promoting the acquisition of confusable spelling rules in primary school children, highlighting its potential to support long-term retention. Furthermore, the results emphasize the added value of instructional guidance in enhancing the long-term transfer of these acquired spelling rules when combined with interleaving. However, the emergence of transfer effects depended on children’s prior knowledge, indicating that instructional guidance alone may not be sufficient to fully support children with low prior knowledge in complex learning tasks. In the present study, brief instructional videos with verbal explanations and examples were provided before the practice phase; however, their effectiveness in enhancing children’s understanding of the spelling rules was not examined. Future research should explore whether providing explicit instruction to build a broader knowledge base prior to the practice phase enables children with low prior knowledge to also benefit from interleaving in terms of transfer. Such an approach could allow instructional guidance to build upon an existing foundation rather than compensate for its absence, thereby maximizing the effectiveness of interleaved practice in promoting rule abstraction and transfer for all learners.

## Data Availability

Additional materials for this article can be found at the repository of the Open Science Framework (https://osf.io/jtzvm). The materials used in this study are available from the authors upon request.

## Footnotes

1 Please note that the hypotheses are numbered differently than in the preregistration for clarity and readability.
